# Regulatory Effect of Mesenchymal Stem Cells on T Cell Phenotypes in Autoimmune Diseases

**DOI:** 10.1155/2021/5583994

**Published:** 2021-03-30

**Authors:** Zhiping Wei, Jintao Yuan, Gaoying Wang, Dickson Kofi Wiredu Ocansey, Zhiwei Xu, Fei Mao

**Affiliations:** ^1^Key Laboratory of Medical Science and Laboratory Medicine of Jiangsu Province, School of Medicine, Jiangsu University, Zhenjiang, 212013 Jiangsu, China; ^2^The People's Hospital of Danyang, Affiliated Danyang Hospital of Nantong University, Zhenjiang, Jiangsu 212300, China; ^3^Directorate of University Health Services, University of Cape Coast, Cape Coast, Ghana

## Abstract

Research on mesenchymal stem cells (MSCs) starts from the earliest assumption that cells derived from the bone marrow have the ability to repair tissues. Several scientists have since documented the crucial role of bone marrow-derived MSCs (BM-MSCs) in processes such as embryonic bone and cartilage formation, adult fracture and tissue repair, and immunomodulatory activities in therapeutic applications. In addition to BM-MSCs, several sources of MSCs have been reported to possess tissue repair and immunoregulatory abilities, making them potential treatment options for many diseases. Therefore, the therapeutic potential of MSCs in various diseases including autoimmune conditions has been explored. In addition to an imbalance of T cell subsets in most patients with autoimmune diseases, they also exhibit complex disease manifestations, overlapping symptoms among diseases, and difficult treatment. MSCs can regulate T cell subsets to restore their immune homeostasis toward disease resolution in autoimmune conditions. This review summarizes the role of MSCs in relieving autoimmune diseases via the regulation of T cell phenotypes.

## 1. Introduction

There are abundant sources of MSCs including the umbilical cord, placenta, bone marrow, adipose tissue, gums, endometrium, menstrual blood, synovium, periosteum, skeletal muscle, and ligamentum cruciatum, among other tissues [1–5]. Besides, human induced pluripotent stem cells (iPS) also serve as a source of MSCs. Although there are variations in the criteria of surface markers of MSC from different sources, the literature shows that classic MSCs express CD105, CD73, CD90, CD34, and CD44, but not CD45, CD34, CD14, CD11b, CD79a, CD19, and HLA-DR [6, 7]. MSCs have the potential of self-renewal and multidirectional differentiation, and their differentiation potential depends on the tissues from which they originate (Table 1). Because of the low immunogenicity and homing capabilities of MSCs, they are used to treat multiple disease conditions. For instance, human umbilical cord-derived mesenchymal stem cells (UC-MSCs) have the potential to transform into cardiomyocyte-like cells. According to Peter et al., the cardiomyocyte-like contractile cells are produced in vitro by MSC differentiation and aggregation on the cardiomyocyte feeder layer and that only the young MSCs could maintain their low immunogenicity after differentiation into cardiomyocyte-like cells [8]. Similarly, Yu and colleagues found UC-MSCs have higher liver differentiation potential than bone marrow MSCs (BM-MSCs), hence their superiority to BM-MSCs in the treatment of end-stage liver disease [9]. This indicates the differential ability of MSCs from varying sources towards specific tissue repair and regeneration.

Also, the proliferation and differentiation potential of MSCs can be enhanced by the culture environment via modification techniques; therefore, the establishment of various modified culture systems makes the application of MSCs in regenerative medicine even more promising. These modification approaches can be roughly divided into genetic modification and preconditioning modification (using drugs, growth factors, and other molecules), which can improve the inherent biological activities concerning migration, homing to target site, adhesion, and survival and reduce premature senescence [10]. Existing research has shown that MSCs communicate with other cells through direct contact and paracrine signaling. In effect, MSCs repair tissue by directly contacting, adhering, and subsequently differentiating into the injured cells. It also exerts its anti-inflammatory, repairing, and immunomodulatory effects by secreting extracellular vesicles (EVs) or paracrine factors and mitochondrial transfer [11].

Autoimmune diseases are caused by imbalanced homeostasis of the autologous environment including T cells. While peripheral regulatory T cell (pTreg) and T helper type 17 (Th17) cell share a common precursor cell (the naïve CD4 T cell) and require a common signal for initial differentiation (tumor growth factor- (TGF-) *β*), they turn to elicit opposite functions via terminal differentiation: Treg is anti-inflammatory, inhibits autoimmunity, and maintains immune homeostasis, whereas Th17 cell causes autoimmunity and inflammation [12]. Moreover, the instability within T cell phenotypes such as Treg alongside their cellular plasticity and tissue-specificity also affects the development of autoimmune diseases [13]. Detailed exploration of T cell interaction with both immune and nonimmune cells presents not only deeper insight into disease pathogenesis but new therapeutic strategies as well.

MSC-based therapy is widely used in refractory immune diseases and has achieved encouraging results. They effectively restore T cell balance within the autoimmune environment, enhancing inflammation resolution through the complemented effect of both cells. This paper examines the application of MSCs in autoimmune diseases such as inflammatory bowel disease (IBD), rheumatoid arthritis (RA), and systemic lupus erythematosus (SLE), among others, and particularly highlights their modulatory effects on T cell phenotypes and the resultant contribution towards therapeutic strategies.

BM-MSCs: bone marrow-derived MSCs; UC-MSCs: umbilical cord-derived MSCs; AD-MSCs: adipose-derived MSCs; DP-MSCs: human dental pulp-derived MSCs; SD-MSCs: synovium-derived MSCs.

## 2. Application of MSCs in Autoimmune Diseases

Autoimmune disease is a diverse kind of complex and heterogeneous abnormal condition caused by immune system disorder. Common examples include IBD, RA, SLE, type 1 diabetes (T1D), and multiple sclerosis (MS), among others. In addition to the difficulties in early detection and poor curative effect, patients with autoimmune diseases are also faced with complicated pathogenesis [30]. At present, due to the low immunogenicity and multidirectional differentiation of MSCs, it is promising to study its therapeutic effect on patients with autoimmune diseases.

### 2.1. Application in IBD

IBD refers to a group of chronic and heterogeneous intestinal inflammatory disorders, including ulcerative colitis (UC) and Crohn's disease (CD). The pathogenesis of IBD is highly complex and not completely clear, but current literature shows that it is related to several factors such as genetic susceptibility, environmental triggers, intestinal flora, diet, psychology, and immunity [31, 32]. The incidence and prevalence of IBD vary from region to region. European countries such as Norway and Germany have the highest global prevalence of 505/100,000 in UC and 322/100,000 in CD, respectively, followed by the North American countries, the United States of America with a UC prevalence of 286/100,000, and Canada with CD prevalence of 319/100,000. Due to the recent modernization of Asian and Latin American countries, the prevalence of IBD in such countries is also constantly rising [33–36].

At present, there are several clinical interventions for IBD patients, including drug therapy such as immunomodulators, steroids, and antibiotics, surgical treatment, and fecal microbiota transplantation (FMT) as a novel therapy. However, these treatment approaches are insufficient in curing IBD. Drug therapies are less effective and often associated with adverse reactions. Surgical procedures have certain requirements for patients' state, and FMT is relatively safe but linked with many problems such as patient acceptance [37–40]. In the phase of these challenges, autologous or allogeneic MSCs emerged as a potentially effective therapy for IBD because of their anti-inflammatory and tissue repair effects, excellent immunomodulatory properties, and low immunogenicity [41]. Experimental studies illustrate that MSCs can repair dextran sulfate sodium- (DSS-) induced acute and chronic colitis in mouse models and prevent the recurrence of experimental IBD. In this process, MSCs regulate immune response, reduce inflammatory cell infiltration, regenerate intestinal epithelial cells, blood vessels, and lymphatic vessels, and change gut microbiota [42–44]. A myriad of clinical trials also shows that MSC therapy is well tolerated with promising efficacy and safety profile. Most recorded adverse effects described for MSCs are mild and transient [45].

A randomized, double-blind, parallel-group, placebo-controlled trial divided 212 patients having a CD with refractory perianal fistula into two groups, which were treated with either autologous AD-MSCs or placebo. The comprehensive remission rates of patients treated with AD-MSCs were higher than those of the placebo group, 50% and 34%, respectively. The incidence of treatment-related adverse events was 17% in AD-MSCs and 29% in placebo groups [46]. Clinical trials by Park et al. also demonstrated the effectiveness of MSCs for perianal fistula repair. A large number of systematic reviews and meta-analyses have also reported the effectiveness and safety of MSCs as IBD-related treatment, especially with long-term effects [47–49]. A study designed to investigate the occurrence of adverse events related to acute infusion toxicity, long-term adverse events, and efficacy of human amnion-derived MSCs (AMSCs) was carried out among CD patients who only achieve partial symptomatic relief with traditional therapy. The results of this phase I/II trial study will be beneficial to further promote the clinical application of AMSCs in IBD [50]. Different doses of BM-MSCs were injected locally in CD patients with refractory perianal fistula to determine the effective dosage that promotes healing of perianal fistulas. The authors concluded that no severe adverse events were associated with the allogeneic MSCs administered and the injection of 3 × 10(7) MSCs appeared to promote healing of perianal fistulas [51].

However, it is important to note that regardless of the success witnessed in MSC therapy in IBD, it is still confronted with several challenges such as severe adverse events, encouraging tumor growth and metastasis, among other reactions as detailed in recent reviews by Ocansey et al. [52, 53]. There is the need to identify supportive or combined therapies of MSC transplantation and also choose the most appropriate stem cell and treatment approach to enhance effectiveness while avoiding the occurrence of serious adverse events [54].

### 2.2. Application in RA

RA is a systemic autoimmune disease, which mainly affects and damages joints and bones. The main clinical manifestations are pain, swelling, deformation of joints, and dyskinesia. In RA, there are unresolved immune cells infiltrating joints and unregulated autoantibody levels. Additionally, RA also affects other organs, including the blood vessels, kidneys, heart, and lungs, resulting in severe pain in patients. The complexity of the definition of RA makes it difficult to study its incidence. According to the criteria defined by the American Rheumatology Society 1987, the global prevalence of RA is about 0.24% (95% CI 0.23% to 0.25%), and the number of patients with RA will reach 4.8 million by 2010 (95% CI 3.7 million to 6.1 million). However, after the revision of the diagnostic criteria of RA in 2010, the number of patients with RA increased further [55, 56]. The main risk factors of RA are gender (women usually have a higher risk than men), heredity, environment, and psychological factors [57]. Recent studies have also shown that RA has a strong correlation with periodontal diseases [58, 59].

At present, there is no effective therapy for RA. The use of nonsteroidal anti-inflammatory drugs and cortisol can alleviate the symptoms of pain and stiffness, but cannot delay the progress of the disease. The use of disease-modifying antirheumatic drugs such as methotrexate and sulfasalazine can delay the disease progression. For example, the depletion of B cells and the development of B cell inhibitory antibodies, IL-6 inhibitors, and T cell-targeted drugs can bring a glimmer of hope for the treatment of RA, but the adverse reactions and toxic side effects of these drugs still hinder their application [60]. Presently, the application of MSCs as a treatment option for RA has demonstrated unique advantages in a host of clinical trials.

According to Wang et al., patients with insufficient response to traditional RA drugs were divided into two groups: patients injected with traditional antirheumatic drugs plus culture medium not containing UC-MSCs and patients injected with traditional antirheumatic drugs and culture medium containing UC-MSCs. They found that the group that received UC-MSCs was significantly relieved, and only one infusion capably achieved the relief effect for 3-6 months, with no major adverse reactions [61]. Another group of researchers selected refractory RA patients in phase Ib/IIa clinical trials and reported similar results without serious side effects in the short term after intravenous injection of AD-MSCs [62]. These demonstrated safety and efficacy studies indicate the encouraging development of MSC therapy in RA, regardless of challenges that demand further exploration in areas such as maintaining the therapeutic effect for a long period.

### 2.3. Application in SLE

SLE, a common autoimmune disease, involves an inflammatory disorder of multiple organs and systems of the body such as the kidney, lung, and skin. It mostly affects females, and some patients develop symmetrical butterfly erythema or other rashes on their faces. The main cause of mortality and morbidity of SLE patients at the end stage is lupus nephropathy [63]. SLE also has the characteristics of complex pathogenesis which largely remains unclear. Notwithstanding, it is agreed that the pathogenesis of SLE is related to the imbalance of factors such as heredity, environment, and endocrine and autoimmune system [64]. Although many genes related to SLE have been found, including complement component-related genes (C1q, C1r, C1s, and C4) and HLA-DR, the specific effects of each gene are still unknown [65, 66]. Besides, estrogen and prolactin have also been identified as risk factors of SLE, which is consistent with the higher prevalence in women than men. Ultraviolet radiation can also increase the risk of SLE. Most SLE patients produce autoantibodies that are related to the clearance defect of apoptotic cells [67]. The global incidence and prevalence of SLE vary with gender, age, race, and time, which are partly explained by the differences in genetic and environmental risk factors [68].

Current treatment of SLE includes immunomodulators and immunosuppressants such as hydroxychloroquine and other drugs that prevent complications. While symptomatic treatment is given to subside various systemic manifestations [69], monoclonal antibody therapy is administered to target and deplete B cells as a proposed treatment of lupus nephritis [70]. Both the traditional and newly developed therapies have limitations related to drug administration, as well as gradual drug reduction until withdrawal, both of which may affect the balance between disease activity control and organ damage caused by long-term and/or unbalanced immunosuppression [71, 72].

MSCs have been used in the treatment of lupus nephritis and refractory SLE patients for more than ten years. Most documented clinical trials are self-controlled studies with only a few being randomized controlled trials. In a meta-analysis study that evaluated the efficacy and safety of MSC treatment in SLE patients, the researchers report that the MSC group showed significantly decreased SLE disease activity index, as well as decreased urine protein, and increased complement C3 [73]. A meta-analysis of the animal model of lupus nephritis also confirmed that MSC treatment resulted in lower levels of disease-associated elements such as double-stranded DNA (ds-DNA), antinuclear antibody (ANA), serum creatinine (Scr), blood urea nitrogen (BUN), proteinuria, and renal sclerosis score, as well as higher albumin levels [53]. Several other studies have reported the promising effect of MSCs in SLE experimental studies and clinical trials [74]. These current pieces of evidence show that MSCs capably improve the disease activity, hypocomplementemia, and proteinuria in SLE patients. However, large-scale and high-quality randomized controlled trials are required to validate the efficacy and safety of MSC treatment in SLE patients. It is also worth noting that allogeneic and autologous MSC treatment of SLE may have opposite effects; hence, allogeneic rather than autologous MSC transplantation could be potentially advantageous for SLE patients [63, 75, 76].

### 2.4. Application in Other Autoimmune Diseases

Recent studies demonstrate the remarkable therapeutic effectiveness of MSCs towards several other autoimmune diseases such as type 1 diabetes [77], multiple sclerosis [78], Hashimoto's autoimmune thyroiditis [79], autoimmune hepatitis, primary biliary cirrhosis [80], and vitiligo [81], among others. For example, MSCs have been shown to prevent inflammation and neurodegeneration in animal models of multiple sclerosis (MS). These experimental studies have set the ground for clinical trials such as a recent randomized, double-blind, placebo-compared phase I/II clinical trial with autologous BM-MSCs in MS which is currently ongoing (ClinicalTrials.gov NCT01854957) [82]. Autoimmune destruction of insulin-producing B cells in the pancreas results in type 1 diabetes, a disease condition that demands more than a mere administration of exogenous insulin to gently and sensitively regulate blood glucose concentration. MSCs can transdifferentiate into insulin-producing cells, support the regeneration of residual B cells via production of growth and trophic factors, or participate in the suppression of the autoimmune reaction against B cells [83, 84]. Hashimoto's thyroiditis (HT) is a disease wherein lymphocytes mediate the autoimmune damage and destruction of the thyroid gland. MSCs have been demonstrated to improve HT via reducing the level of thyroid autoantibody partly by regulating Th17/Treg interactions [79].

A detailed exposition of research progress on MSC therapy in autoimmune diseases indicating remarkable therapeutic effectiveness has recently been reviewed by Chen and colleagues [85]. MSCs also capably home to the disease site, regulating the balance of T cells through direct contact and secretion of active factors.  Table 2 presents some of the documented studies of MSC's role in immune regulation of selected autoimmune diseases.

T1D: type 1 diabetes mellitus; TGF-*β*/MSCs: TGF-*β* engineered MSCs; MS: multiple sclerosis; SS: Sjögren's syndrome; PBC: primary biliary cirrhosis; HT: Hashimoto's thyroiditis.

## 3. Regulatory Effect of MSCs on T Cells in IBD

In the experimental IBD model, MSCs regulate the generation of T cell subsets to alleviate intestinal inflammation [102, 103]. For instance, the coculture of peripheral blood mononuclear cells (PBMCs) and MSCs strongly inhibits the proliferation of CD4+ and CD8+ T cells, as well as natural killer (NK) cells. The researchers found that the mechanism involved is not dependent on cell contact, but rather activated by interferon-gamma (IFN-*γ*) produced by lymphocytes. IFN-*γ* stimulates MSCs to produce indoleamine 2,3-dioxygenase (IDO), prostaglandin E2 (PGE2), and IL-10, wherein the IDO principally inhibits the proliferation of T lymphocytes [104, 105]. MSCs homing in colon tissue can promote the proliferation of intestinal epithelial cells and the regeneration of intestinal stem cells. This effect has been shown to be related to the downregulation of Th1/Th17. Other molecules reduced in the process owing to the anti-inflammatory effect of IFN-*γ* include interleukin- (IL-) 2, tumor necrosis factor- (TNF-) *α*, IFN-*γ*, T-bet, IL-6, IL-17, and retinoic-acid-receptor-related orphan nuclear receptor gamma (ROR*γ*t) [106]. However, in most cases, IFN-*γ* is still considered a proinflammatory factor. It has been reported that MSCs significantly inhibit the secretion of IFN-*γ* and promote the production of IL-10 by T cells. IL-10 acts with dendritic cells (DCs) to promote anti-inflammatory effect [107, 108]. These findings show the complex role of IFN-*γ* in MSC-mediated immune regulation including its role in inducing T cell inhibition via MSC regulation.

Regulatory T cells (Tregs), the special T cell subset for immunosuppression, specifically express transcription factor forkhead box P3 (FoxP3) in the nucleus and CD25 and CTLA-4 (cytotoxic T lymphocyte-associated protein 4) on the cell surface [109]. Tregs play a crucial role in the inhibition of inflammation associated with several diseases such as IBD [110]. The combination of MSCs and Tregs in experimental treatment results in longer survival time for exogenous Tregs, upregulation of endogenous Tregs, and downregulation of proinflammatory Th17 cells [111, 112]. It is worth noting that endogenous CD4+CD25+Foxp3+ T cells are differentiated from CD4+ T cells, rather than natural Treg amplification, which involves TGF-*β* and/or programmed cell death- (PD-) 1/PD-L1 mechanism [45, 105].

Studies by Sarah and others indicate that cytokines secreted by MSCs intensely participate in the immunomodulatory role on T cells. TGF-*β*1, a soluble cytokine produced by MSCs, can induce Tregs under TCR (T cell receptor) costimulation, promote the activation of monocytes, and enhance monocyte differentiation into type II macrophages. Macrophages produce a large amount of IL-10 and CCL-18 (C-C motif chemokine ligand-18), which has been shown to play an important role in Treg induction; IL-10 further inhibits the pathogenicity of Th17 [113]. Macrophages can significantly inhibit the proliferation of CD4+ T cells and reduce the content of inflammatory factors TNF-*α* and IFN-*γ* [114]. TGF-*β* can also induce Foxp3 expression, inhibit Th17 differentiation, and stimulate Treg development [103, 115], via its biological activities through transcription regulation of several genes. Activated TGF-*β* binds to TGF-*β*1 and TGF-*β*2 receptors, followed by induction of the formation of phosphor-mothers against decapentaplegic homolog 2 (pSmad2), pSmad3, and Smad4 complexes, thus activating the intracellular signal activation of TGF-*β* signaling [105, 116]. Studies have shown that the lack of TGF-*β*1 leads to severe colonic inflammation, while the restoration of TGF-*β*1 activity improves the resolution of colitis [117, 118]. MSCs can block the induction of inflammatory-associated TNF and interleukins while promoting T cells to secrete anti-inflammatory cytokine like IL-10. The secretion of polyethylene glycol (PEG) by MSCs in the process of inflammation resolution has also increased significantly, as PEG is an important regulator to maintain immune homeostasis [119].

The immunomodulatory effect of MSCs is not only due to the role of soluble factors but also the effect of intercellular contact. MSCs constitutively express FasL and PD-L1. FasL induces apoptosis of activated T cells, while PD-L1 on the surfaces of MSCs combine with PD-1 on the surfaces of T cells, exerting immunosuppression through major histocompatibility complex II (MHC II). MSCs can also secrete IFN-*β* to increase the expression of PD-1 on the surface of T cells and strengthen the inhibition of T cells [105, 107, 120]. Mice with PD-1 gene knocked out produce autoimmune diseases, which also prove that MSCs can inhibit the activation, expansion, and cytokine production of T cells through the PD-1/PD-L1 pathway [121]. Experiments prove that tonsil-derived MSCs (T-MSCs) weaken the differentiation of Th17 and directly regulate the phosphorylation of signal transducer and activator of transcription 3 (STAT3) through the PD-L1 expression [122]. The imbalance of the IL6/IL6R-STAT3-SOCS3 (suppressor of cytokine signaling 3) pathway is closely related to IBD-related diseases [123, 124]. MSCs express NOD2 (nucleotide-binding oligomerization domain-containing protein 2), and its binding with ligand MDP (muramyl dipeptide) enhances the production of PEG2 and increases the production of IL-10 and Tregs through NOD2-RIP2 (receptor-interacting protein 2) pathway. In several experimental colitis models in mice, MSCs have been demonstrated to highly express Jagged-1, induce Notch signaling of T lymphocytes, reduce the activity of NF-*κ*B, reduce the production of IL-2 and IFN-*γ*, and hinder the proliferation of T lymphocytes [125–128].

Intercellular adhesion molecule-1 (ICAM-1), also known as CD54, is involved in signal transmission between cells, regulates immune response, and mediates cell differentiation, development related to lymphocyte homing and circulation. Generally, ICAM-1 is not expressed on the surface of MSCs, but ICAM-1 is upregulated in the inflammatory microenvironment. MSCs overexpressing ICAM-1 significantly reduced the percentage of Th1 and Th17 cells in the spleen, increasing the number of Tregs of IBD mice. Further analysis revealed remarkably reduced mRNA levels of INF-*γ* and IL-17A and promoted expression of Foxp3, thus alleviating the experimental colitis [129].

Nitric oxide (NO) produced by MSCs can also inhibit the expression of CD25 in T cells by regulating LKB1- (liver kinase B1-) AMPK- (adenosine 5′ monophosphate-activated protein kinase-) mTOR pathway. It is reported that the deletion of LKB1 decreases AMPK phosphorylation level and activates mTORC1, which leads to T cell activation and inflammation, while MSCs can increase LKB1 and AMPK phosphorylation level, thus exerting inhibitory effects on inflammatory T cell proliferation and increasing anti-inflammation [130]. IL-25 can inhibit TNF-*γ* and IL-17A produced by CD4+ T cells of IBD patients, promote the secretion of anti-inflammatory IL-10, and inhibit the differentiation of CD4+ T cells of IBD into proinflammatory Th1 and Th17 cells [131, 132].

It is worth mentioning that peripheral circulating T lymphocytes play an important role in the MSC treatment mechanism of IBD. There are groups of intestinal intraepithelial lymphocytes (IELs) that are similar to peripheral lymphocytes in the intestinal tract and have complex cell subsets, including TCR-positive and TCR-negative cells. Every ten intestinal epithelial cells (IECs) in the small intestine contain about one IEL, which is lower in the colon. IELs reside in intestinal epithelial cells and do not participate in the circulation. They are associated with the maintenance of the intestinal mucosal immune barrier [133, 134]. The increase of IELs can be observed in the intestinal tract of children with IBD [135]. The imbalance of IEL subsets is related to the pathogenesis of IBD, but it is still unknown whether MSCs can regulate IELs.

**Figure 1:**
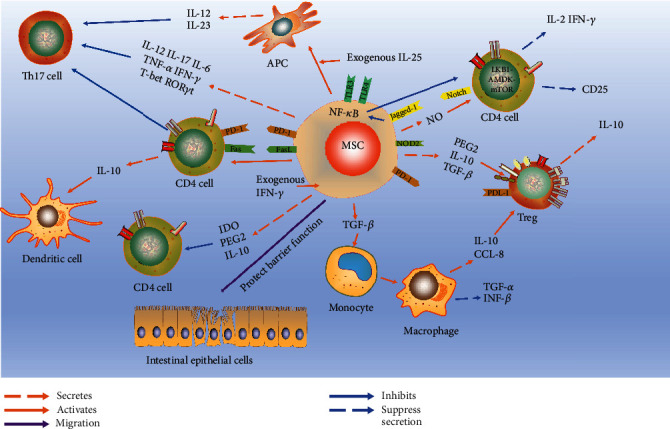
MSCs alleviate IBD by regulating T cells. MSCs induce CD4 T cells to differentiate into Treg and maintain Th17/Th1 balance through a series of cytokines and cell-to-cell contact. This results in decreased inflammatory activities to repair intestinal inflammation.

IDO plays an important role in the regulation of MSCs on experimental enteritis in mice. MSCs can secrete IDO, which is a rate-limiting enzyme that catalyzes tryptophan metabolism. IDO and its downstream metabolites kynurenine (KYN) and kynurenic acid (KYNA) play a powerful role in inhibiting T cell proliferation and Treg differentiation [136, 137]. IDO can alleviate DSS-induced enteritis by regulating tryptophan metabolites KYN and KYNA in MSCs, activating transcription factor aryl hydrocarbon receptor (AhR), and upregulating the expression of TNF-stimulated gene 6 (TSG-6) [138]. Under the action of the inflammatory microenvironment, MSCs enhance the glycolytic pathway and upregulate the IDO level through the Janus kinase (JAK)/STAT1 pathway, which plays an immunosuppressive role [139, 140]. The activities of MSCs in IBD as discussed above are summarized in Figure 1.

## 4. Regulatory Effect of MSCs on T Cells in RA

The mechanism by which MSCs regulate T cells to relieve RA overlaps with the mechanism of regulating IBD. Just as reported in other autoimmune diseases, there is an imbalance of T cell subsets in RA patients too, including Th17/Treg cells which are capably regulated by MSCs. The expression of TNF-*α* inducible protein 3 (TNFAIP3), also known as A20, from BM-MSCs of RA patients has been found to be reduced. TNFAIP3 is a protective protein of chronic arthritis, which can negatively regulate the NF-*κ*B pathway and reduce the expression of IL-6. MSCs overexpressing A20 can inhibit the expression of IL-6, thus restoring Th17/Treg balance. A20 deficiency also increases Th17 and decreases Tregs, while Th1 and Th2 are not affected. Specifically, inflammatory cytokines induce A20 expression in MSCs. Mechanism studies show that knocking out A20 in MSCs can inhibit the activation of the p38 mitogen-activated protein kinase (MAPK) pathway, effectively promote the production of TNF-*α*, and inhibit the production of IL-10. It is worth noting that the therapeutic effect of MSCs on RA may be different due to the different expression of A20 [76, 141]. Mitochondrial transfer from MSCs to Th17 cells, as a mechanism of MSC regulating immunity, can occur through intercellular contact, resulting in increased oxygen consumption of Th17 and reduced production of IL-17. At the same time, the mitochondria of Treg markers on the surface of Th17 cells increase, indicating that mitochondria from MSCs can increase the production of anti-inflammatory phenotype [142]. Inflammatory microenvironment can lead to the activation of the PI3K/Akt/mTORC1 pathway, which is closely related to cell metabolism and can enhance glycolysis and activate lymphocytes. Mitochondrial metastasis can transform energy metabolism into oxidative phosphorylation, and Treg-related markers are upregulated while proinflammatory markers are downregulated [143, 144].

In addition to Th17 cells, T follicular helper (Tfh)/T follicular regulatory (Tfr) cells are also closely related to RA. It is reported that the number of Tfh and Tfr in RA patients is increased, but the ratio of Tfr/Tfh is decreased, with a significantly increased number of circulating B cells related to Tfh [145, 146]. The production of autoantibodies in RA patients, such as antirheumatoid factor (RF) and anticyclic citrullinated peptide (CCP), leads to the deposition of immune complexes, while Tfh cells can migrate to the germinal center (GC) to maintain the differentiation of B cells. Tfh is closely related to the production of autoantibodies in B cells. Tfh cells express high levels of CXCR5, PD-1, IL-21, and other characteristic markers, and their cellular differentiation is regulated by a complex network of transcription factors, including positive factors (Bcl6, ATF-3, Batf, IRF4, c-Maf, etc.) and negative factors (Blimp-1, STAT5, IRF8, Bach2, etc.) [147, 148]. On the other hand, Tfr is a type of cell in the Treg subgroup, which can inhibit the reaction in GC and the production of high-affinity antibody. As an inflammatory factor, IL-6 plays a role in the pathogenesis of RA, by phosphorylating STAT3 and participating in Tfh differentiation [149]. MSCs can significantly reduce the production of IL-6 in vivo, which may have an alleviating effect by regulating Tfh/Tfr. Whether MSCs can regulate these transcription factors and participate in the regulation of Tfh cells in RA patients is rarely reported at present, which is also the direction of future research.

MSCs can exert their immune function and relieve autoimmune diseases through PEG2, TGF-*β*, HGF, IL-10, and IDO, which are found in RA, IBD, and other autoimmune diseases. Studies have shown that endoplasmic reticulum- (ER-) stressed MSCs can produce higher levels of IL-10 and PEG2 than ordinary MSCs and downregulate CD4+CXCR5+ICOS+ T cells (Tfh) in RA patients. It may be that glucose-regulated protein 78 (GRP78) and X-box binding protein 1 (XBP-1) are strongly induced in ER-stressed MSCs, resulting in a large amount of PEG2 production. PEG2 receptors, membrane-bound G protein-coupled receptors termed EP1, EP2, EP3, and EP4, are expressed on CD4+ T cell surface. PEG2 can also increase the levels of IL-12 and IFN-*γ*, which triggers Th1 cells to differentiate. The anti-T cell proliferation effect is realized by the EP/COX2/PEG2 axis, wherein COX2 is upregulated under the condition of inflammatory stress [150–152].

In RA patients, MSCs can phagocytize apoptotic cells (ACs) in an actin-dependent way and secrete IL-6, IL-17, and RANTES (regulated upon activation, normal T cell expressed and presumably secreted). In this process, MSCs can express CXCR4, CXCR5, and ICAM-1 and migrate to inflammatory joints through the SDF/CXCR4 pathway, which makes synovial CD4+CD25+CD69+ T cells increase and makes them express IL-17, FoxP3, and RANKL, known to promote the increase of osteoclasts. Th17 cell differentiation depends on IL-6 and IL-1*β*, and intercellular contact mediated by costimulatory molecules CD25, ICOS (inducible costimulatory), and TL1A (TNF-like ligand 1A) can also participate in Th17 cell differentiation. The MSCs induced by RA can express IL-6 and MHCII and then increase the level of IL-17. Cytokines and intercellular contact promote Th17 cells and osteoclast formation. In other studies, RA-induced MSCs did not change the number of CD4+FOXP3+Treg; therefore, MSCs may enhance the pathogenic effect of RA in patients; hence, MSC therapy for RA should be carefully considered [153, 154].

**Figure 2:**
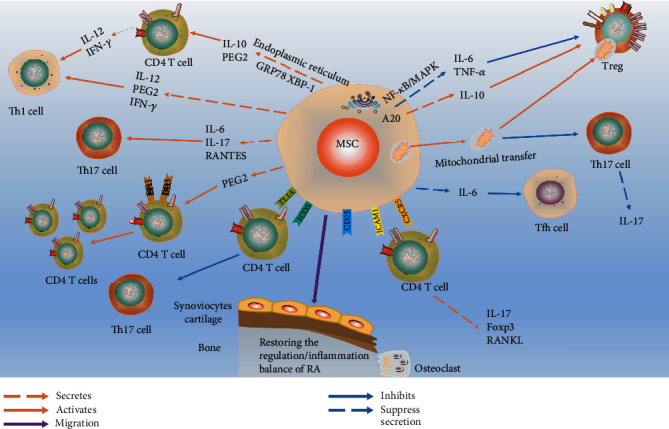
MSCs relieve RA by regulating T cells. MSCs can regulate the balance of T cells by homing to the articular cavity and secreting a series of cytokines that increase the anti-inflammatory activity of the environment. T cells are also regulated via the transfer of mitochondria from MSCs to T cells. Additionally, MSCs under endoplasmic reticulum stress can also play a regulatory role in inducing T cells.

CD4+ T cells expressed by the granulocyte-macrophage colony-stimulating factor (GM-CSF) play certain roles in RA induction. AD-MSCs can reduce the number of GM-CSF+CD4+ T cells. MSCs participate in regulating immune response by promoting early adaptive regulatory T cell signals, which is characterized by a decrease in the level of T cells secreting pathogenic GM-CSF, an increase in the number of Tregs, and the development of effector Th17 cells towards IL-10-driven anti-inflammatory response, thus restoring the regulation/inflammation balance of RA [155]. The effects of MSCs within the RA environment are illustrated in Figure 2.

## 5. Regulatory Effect of MSCs on T Cells in SLE

Literature indicates that the disorder of AC clearance mechanism may be one of the pathogenesis of SLE [67]. It has been demonstrated that MSCs phagocytize ACs and regulate immune homeostasis in vivo [154], in a time- and dose-dependent manner. MSCs exposed to ACs activate the NF-*κ*B pathway by recognizing phosphatidylserine, which leads to highly expressed COX2, associated with the production of a large amount of PEG2. MSCs activated by ACs can also inhibit the proliferation of CD4+ T cells more strongly than controls, which are related to soluble cytokines IFN-*γ* and IL-17. However, how ACs activate the NF-*κ*B pathway in MSCs is still unknown.

The decreased expression of CD4, CD25, and Foxp3 indicates a reduced number of Tregs in SLE patients. While MSCs recover Tregs through secretion of TGF-*β*, Tregs inhibit the response and the production of autoantibodies by B cells through the induction of apoptosis related to the expression of granzyme A, granzyme B, and perforin [156, 157].

Abnormal methylation of T cells in SLE patients leads to overexpression of methylation-sensitive autoimmune genes CD70, ITGAL (integrin subunit alpha L) (CD11a), selectin-1, IL-4, and IL-13 in lupus. CD70, a costimulatory factor, activates B cell response. ITGAL is related to the self-activation of T cells. Studies have shown that MEK/ERK pathway defects and T cell methylation changes in SLE patients lead to increased immune disorders. The MEK/ERK pathway of PBMC from SLE patients can be active after coculture with BM-MSCs. After coculture, DNA methyltransferase 1 DNMT1 was upregulated, and CD7, CD70, integrin, ITGAL, selectin-1, and IL-13 were downregulated in PBMC of patients. BM-MSCs downregulate the expression of methylation-related genes and reduce the self-activation of PBMC through the MEK/ERK pathway [158–160].

Several other studies have investigated the Th17 and Treg imbalance found in SLE patients. Stress response and immune regulation molecule heme oxygenase-1 (HO-1) are involved in the induction of Tregs. MSCs can express HO-1 and participate in the induction of Treg cell subsets, but there are big individual differences. Perhaps HO-1 expressed by MSCs can play certain roles in SLE patients, and the specific mechanism needs to be studied [161, 162].

## 6. Conclusion

As a cell-based therapy, MSCs possess the potential of ameliorating injury or possibly offering a cure for patients with immune-mediated conditions. Available document on the contribution of MSCs in restoring T cell balance within the autoimmune environment is promising, as the MSCs exert their immunoregulatory effects via direct contact and secretion of active factors. The mechanism of action of MSCs overlaps and has quite a few differences in various autoimmune diseases, which may be related to the origin of MSCs and the heterogeneity of autoimmune diseases. Additionally, MSCs treated with certain factors to overexpress desired cytokines result in stronger regulation of T cell immunity. Further explorations of key targets of MSCs during T cell regulation and their associated mechanisms in autoimmune diseases are needed to enhance understanding towards improving the therapeutics of MSCs.

## Figures and Tables

**Table 1:** The surface markers and differentiation potential of different kinds of MSCs.

Sources	Surface marker	Differentiation potential	Reference
BM-MSCs	CD271(+), CD59(+), CD81(+), CD47(+), CD151(+), CD147(+), CD98(+), CD143(-), Lin (-) CD45(-), CD140a (PDGFR*α*) (low/-)	Strong adipogenic and osteogenic potential, poor chondrogenic potential, strong differentiation potential of corneal epithelial cells, and cardiac progenitors	[14–18]
UC-MSCs	CD73(+), CD90(+), CD105(+), CD44(+), CDH-1(+), CD29(+), CD34(-), CD45(-)	Muscle, neurogenic cells, hepatocyte-like cells, endothelial lineage	[19–23]
AD-MSCs	CD45(-), HLA-DR(-), CD44(+), CD106(+), CD34(+), CD90(+), CD105(+)	Strong adipogenic and osteogenic potential, poor chondrogenic potential, poor differentiation potential of corneal and muscle	[14, 16, 17, 24–26]
DP-MSCs	TRO-1(+), CD146(+), CD29(+), CD90(+), CD105(+), CD44(+), CD59(+), CD73(+), CD146(-), CD34(-), CD45(-), CD11b(-), CD45(-)	Osteogenic, adipogenic, chondrogenic, fibroblast lineage, neural stem cells	[27, 28]
SD-MSCs	CD9(+), CD10(+), CD13(+), CD44(+), CD54(+), CD55(+), CD90(+), CD105(+), CD166(+), D7-FIB(+), CD14(-), CD20(-), CD45(-), CD133(-)	Strong chondrocyte, osteocyte, and adipocyte differentiation ability, as well as muscle differentiation	[16, 29]

**Table 2:** The application of MSCs in other autoimmune diseases.

Disease	Source of MSCs	Effects	Reference
T1D	UC-MSCs TGF-*β*/MSCs	MSCs were safe and tolerable Hyperglycemia was significantly controlled	[77, 86–89]
MS	BM-MSCs	Clinically feasible and relatively safe and could immediately produce immune regulation	[90–94]
SS	UC-MSCs	Effective in treatment	[95, 96]
PBC	BM-MSCs UC-MSCs	MSCs were well tolerated and no obvious side effects were found Symptoms were significantly alleviated	[97–99]
HT	AD-MSCs	MSCs inhibited inflammation and helped recover from injury	[79, 100, 101]
